# Quantifying tissue properties and absolute hemodynamics using coherent spatial imaging

**DOI:** 10.1117/1.JBO.28.12.127001

**Published:** 2023-12-19

**Authors:** Christian Crouzet, Cody E. Dunn, Bernard Choi

**Affiliations:** a University of California, Irvine, Beckman Laser Institute and Medical Clinic, Irvine, California, United States; b University of California, Irvine, Department of Biomedical Engineering, Irvine, California, United States; c University of California, Irvine, Department of Surgery, Irvine, California, United States; d University of California, Irvine, Edwards Lifesciences Foundation Cardiovascular Innovation Research Center, California, United States

**Keywords:** optical properties, blood flow, hemoglobin concentrations, diffuse optics

## Abstract

**Significance:**

Measuring hemodynamic function is crucial for health assessment. Optical signals provide relative hemoglobin concentration changes, but absolute measurements require costly, bulky technology. Speckleplethysmography (SPG) uses coherent light to detect speckle fluctuations. Combining SPG with multispectral measurements may provide important physiological information on blood flow and absolute hemoglobin concentration.

**Aim:**

To develop an affordable optical technology to measure tissue absorption, scattering, hemoglobin concentrations, tissue oxygen saturation (${\mathrm{StO}}_{2}$), and blood flow.

**Approach:**

We integrated reflectance spectroscopy and laser speckle imaging to create coherent spatial imaging (CSI). CSI was validated against spatial frequency domain imaging (SFDI) using phantom-based measurements. *In vivo* arterial and venous occlusion experiments compared CSI with diffuse optical spectroscopy/diffuse correlation spectroscopy (DOS/DCS) measurements.

**Results:**

CSI and SFDI agreed on tissue absorption and scattering in phantom tests. CSI and DOS/DCS showed similar trends and agreement in arterial occlusion results. During venous occlusion, both uncorrected and corrected blood flow decreased with increasing pressure, with an ∼200% difference in overall blood flow decrease between the methods. CSI and DOS/DCS data showed expected hemoglobin concentrations, ${\mathrm{StO}}_{2}$, and blood flow trends.

**Conclusions:**

CSI provides affordable and comprehensive hemodynamic information. It can potentially detect dysfunction and improve measurements, such as blood pressure, $S_{p}O_{2}$, and metabolism.

## 1 Introduction

Optical signals use absorption contrast to quantify hemodynamic information, such as the photoplethysmogram (PPG), which detects absorption changes due to variations in blood volume with each heart contraction. PPG is commonly used in pulse oximetry to estimate arterial blood oxygen saturation ($S_{a}O_{2}$).^1^^,^^2^ Pulse oximetry has become the primary optical technique for continuous patient monitoring in various settings, such as the intensive care unit^3^^,^^4^ and the emergency room.^5^ However, PPG’s accuracy is limited during hypothermia, circulatory shock, motion, and in the presence of melanin.^6^ Speckleplethysmography (SPG) is a new technology that uses laser speckle imaging (LSI) to examine pulsatile blood flow and may overcome the limitations associated with traditional pulse oximetry.^7^^–^^9^ We previously demonstrated that the SPG signal at a single wavelength has an signal-to-noise ratio (SNR) ∼40 times greater than the PPG signal and readily resolves the pulsatile blood flow waveform.^10^ Furthermore, the SPG signal is more resilient to the effects of epidermal melanin than the PPG signal.^11^^,^^12^ Incorporating SPG into devices with a pulse oximetry format could provide blood flow information. However, tissue optical properties can affect LSI, and hence SPG’s accuracy.^13^^,^^14^ Incorporating optical property measurements with LSI may produce a more quantitative blood flow measurement.^15^^,^^16^

In addition, optical techniques can measure relative hemoglobin concentrations using relative absorption changes from light, but cannot quantify absolute hemoglobin concentrations without making assumptions about the tissue scattering and differential path factor. These assumptions can drastically influence absolute measurements of blood volume and tissue oxygen saturation (${\mathrm{StO}}_{2}$).^17^ Technological advancements, such as time-resolved near-infrared spectroscopy (NIRS),^18^ frequency-domain NIRS,^19^ and spatial frequency domain imaging (SFDI),^20^ can account for optical properties but are typically expensive and bulky. Spatially resolved diffuse reflectance spectroscopy (srDRS) exploits point illumination and reflectance measurements at multiple source–detector separations to provide quantitative estimates of optical properties and can measure absolute hemoglobin concentrations and ${\mathrm{StO}}_{2}$.^21^ There are two primary form factors for srDRS: contact fiber probe^21^ and non-contact camera sensors.^22^ The former has limited source–detector separations due to using individual optical fibers. The latter enables high spatial resolution measurements, and with recent miniaturization in component size, it offers the possibility of integration into suitably small devices. A recent low-cost, wearable device uses srDRS principles to quantify tissue optical properties using a camera-based sensor.^23^^,^^24^ This approach enables the measurement of absolute hemoglobin concentrations but not quantitative blood flow. Combining srDRS and LSI can overcome this limitation and provide optical property-corrected blood flow measurements.^15^^,^^16^

In this work, we aimed to measure absolute tissue absorption ($\mu_{a}$), tissue scattering ($\mu_{s}^{\prime }$), oxy- ($\left[{\mathrm{HbO}}_{2}\right]$) and deoxy-hemoglobin ([Hb]) concentrations, ${\mathrm{StO}}_{2}$, and blood flow using a low-cost device with a footprint similar to pulse oximeters to provide additional quantifiable metrics that pulse oximetry does not measure. We achieved this aim by creating coherent spatial imaging (CSI), which combines srDRS and LSI. CSI was validated through phantom-based optical property measurements and *in vivo* arterial and venous occlusions.

## 2 Materials and Methods

**Fig. 1:**
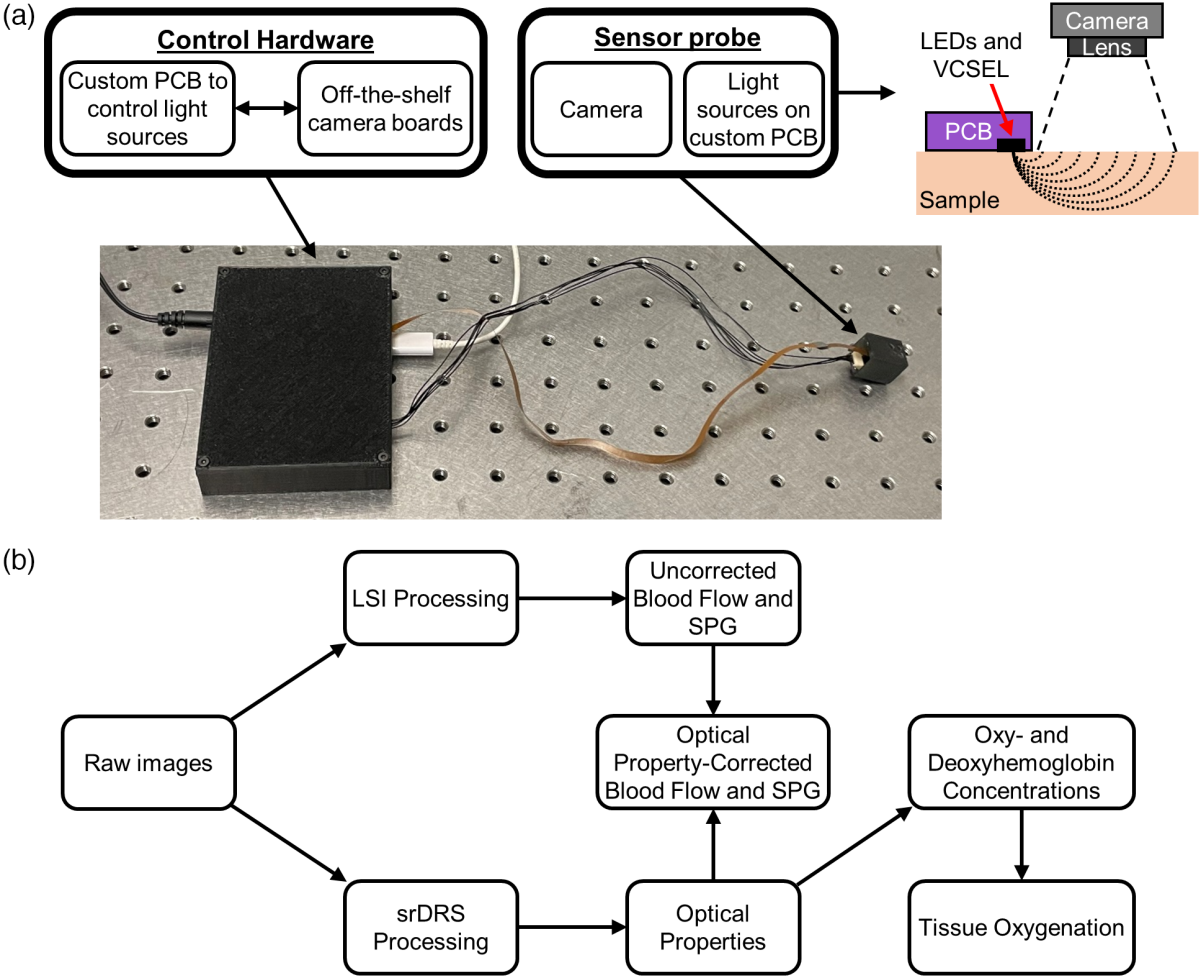
CSI device and processing overview. (a) A custom PCB was developed to control the synchronization between light sources and the camera. The custom PCB was connected to a sensor probe that contains the light sources (LEDs and VCSEL) on a PCB and a camera. The sensor probe was attached to the measured area. The reflected light from the LEDs and VCSEL was collected at source–detector separations of 3 to 10 mm. (b) Processing overview from raw images to absolute quantifiable metrics, including optical properties, blood flow, oxy- and deoxy-hemoglobin concentrations, and tissue oxygenation.

We developed a first-generation CSI device by combining srDRS and LSI principles. The device [Fig. 1(a)] comprises of (1) control hardware with an off-the-shelf camera shield (Arducam, Hong Kong) and a custom printed circuit board (PCB) to control the light source circuitry and (2) a sensor probe with a camera sensor and custom PCB with light sources. The sensor probe measures 20  mm×18  mm×16  mm and employs three wavelengths (660, 740, and 850 nm) to distinguish $\left[{\mathrm{HbO}}_{2}\right]$ and [Hb]. The 850 nm wavelength is a VCSEL to measure SPG and blood flow using LSI. The frame rate of the camera was set to 135 Hz. This resulted in frame rates of ∼34  Hz for each LED and ∼67  Hz for the VCSEL.

Figure 1(b) shows an overview of the data processing steps. A raw CSI image undergoes LSI and srDRS processing. For LSI, a 7×7 sliding window was used to calculate the spatial speckle contrast (K), and a region of interest (ROI) was selected using pixels ranging from source–detector separations of 3.1 to 4.4 mm to obtain the SPG signal. The uncorrected blood flow was defined as $1/K^{2}$. srDRS processing was performed similarly to previous works.^23^^,^^24^ Briefly, raw reflectance as a function of source–detector separation (3 to 10 mm) was obtained from a sample with unknown optical properties and a calibration phantom with known optical properties. A dark image was subtracted from the raw reflectance of the sample and calibration phantom, and a lookup table with theoretical diffuse reflectance curves for each set of unique $\mu_{a}$ (0 to $0.4\;{\mathrm{mm}}^{-1}$) and $\mu_{s}^{\prime }$ (0.3 to $3.5\;{\mathrm{mm}}^{-1}$) values was created using a software from the Virtual Photonics Initiative^25^ at the Beckman Laser Institute. The theoretical diffuse reflectance curve from the lookup table was divided by the reflectance from the calibration phantom to calculate a scale factor. The scale factor was multiplied with the reflectance curves with unknown properties to obtain a calibrated diffuse reflectance. To obtain a final set of optical properties, all sets of optical properties with an $R^{2}>0.98$ for 660 and 740 nm and 0.94 (*in vitro*) and 0.98 (*in vivo*) for 850 nm between the calibrated diffuse reflectance and theoretical diffuse reflectance were averaged together. This averaging approach was used due to several $R^{2}$ values indicating good fits ($R^{2}>0.98$), and we chose to use a conservative approach of averaging multiple sets of data with good fits instead of relying on a single fit that may have an intrinsic error or selection bias. *In vivo* measurements used scatter-corrected absorption to determine hemoglobin concentrations and ${\mathrm{StO}}_{2}$. The combination of optical properties from srDRS and blood flow from LSI allowed for optical property-corrected blood flow measurements using the Brownian diffusion coefficient ($D_{b}$).^13^^,^^16^

We validated the CSI device using both *in vitro* and *in vivo* experiments. We conducted three *in vitro* experiments to assess the ability of CSI to measure optical properties. (1) To assess short-term stability, we imaged a tissue-simulating phantom with CSI over 1-h for 3 days. (2) To evaluate device repeatability over a wide range of optical properties, we imaged 18 silicone-based tissue phantoms with CSI three times over a week. (3) To assess long-term device repeatability, we imaged the same 18 silicone-based tissue phantoms nine times over 3 months. For all *in vitro* experiments, we calculated the coefficient of variation (COV) to determine device repeatability. In *in vitro* experiments 2 and 3, we used SFDI^20^ as the gold standard to compare the optical properties measured by CSI and SFDI. SFDI measurements were carried out using a commercial device (ReflectRS, Modulim, Irvine, California). They involved structured illumination at five sinusoidal spatial frequencies evenly spaced between 0 and $0.2\;{\mathrm{mm}}^{-1}$ at eight wavelengths (471, 526, 591, 621, 659, 691, 731, and 851 nm).^26^ Raw reflectance images were calibrated against images of the calibration phantom, and CSI and SFDI measurements were calibrated against the same phantom. We used a Monte Carlo-based transport forward model on a semi-infinite medium with homogeneous optical properties throughout the imaged tissue volume. We compared CSI-measured optical properties at 660, 740, and 850 nm with SFDI-measured optical properties at 659, 731, and 851 nm.

We validated CSI’s ability to monitor absolute hemoglobin concentrations, ${\mathrm{StO}}_{2}$, and blood flow compared with DOS/DCS during *in vivo* venous and arterial occlusions on human subjects. We compared CSI with DOS/DCS due to the similarities in the output data parameters. However, we acknowledge inherent differences in the optics, the sample volume interrogated, and the measurement locations. Nonetheless, we compared CSI and DOS/DCS to determine whether CSI followed similar trends to DOS/DCS. DOS/DCS measurements used a previously described lab-grade device.^27^ DOS measured tissue optical properties and absolute hemoglobin concentrations. Water absorption was not taken into account. Three laser diodes (727, 808, and 839 nm) were used. A network analyzer (Copper Mountain Technologies, Indianapolis, Indiana) modulated each laser diode from 50 to 300 MHz. An avalanche photodiode (Hamamatsu Photonics, Japan) detected the phase and amplitude at each modulation frequency. The collected data were fit to a light-propagation model to extract the $\mu_{a}$ and $\mu_{s}^{\prime }$. The scatter-corrected $\mu_{a}$ was used to calculate $\left[{\mathrm{HbO}}_{2}\right]$ and [Hb]. The final processed DOS data were measured at 4.9 Hz. DCS measured blood flow using a long coherence length (>20  m) 785 nm laser (CrystaLaser, Nevada). The signal was detected with four single photon counting modules (Excelitas Technologies, Massachusetts). The digital output of each detector was connected to an eight-channel counter/timer data acquisition board to sample the digital pulses generated by the detectors. A real-time DCS software correlator fits the collected data to a mathematical model of dynamic light scattering to measure a blood flow index (BFI). BFI data from DCS were measured at 20 Hz. DOS and DCS source and detector fibers were combined into a single probe with a 15 mm source–detector separation. To mitigate crosstalk between DOS optical sources and DCS detectors, a 785 nm bandpass filter was placed in front of the DCS detector fiber bundle. In addition to comparing CSI with DOS/DCS, we also compared uncorrected and optical-property corrected blood flow within CSI during venous occlusion.

For *in vivo* measurements, each of the three subjects is tested with the CSI probe attached to the finger and the DOS/DCS probe attached to the thenar muscle on the palm. Arterial occlusion was induced using an approved IRB protocol (#20206221) and a mechanically controlled occlusion cuff (Hokanson, Bellevue, Washington) placed over the brachial artery. Informed consent was obtained from all subjects participating in this study. The procedure involved a 2-min baseline period at 0 mmHg, followed by 2 min of full occlusion at 220 mmHg, and then 2 min of recovery at 0 mmHg. Venous occlusion was induced using a previously described procedure,^28^ involving a 2-min baseline period at 0 mmHg, followed by a stepwise venous occlusion at 20, 40, 60, 80, and 100 mmHg, each lasting 1 min, and then a 1-min recovery period at 0 mmHg.

## 3 Results and Discussion

To test the short-term stability of CSI-measured optical properties, we imaged a tissue-simulating phantom over 1 h on 3 different days. COVs for reflectance from LEDs (660 and 740 nm) were <0.4% and from the VCSEL (850 nm) <1.2%. COVs for $\mu_{a}$ from LEDs were <0.9% and from the VCSEL <2.7%. COVs for $\mu_{s}^{\prime }$ from LEDs were <0.6% and from the VCSEL <2.9%. The higher COVs for the VCSEL may be attributed to speckles adding noise to the srDRS curves. The mean COV of the reflectance, $\mu_{a}$, and $\mu_{s}^{\prime }$ from all wavelengths was 0.78%, indicating the device’s stability for a 1-h measurement.

**Fig. 2:**
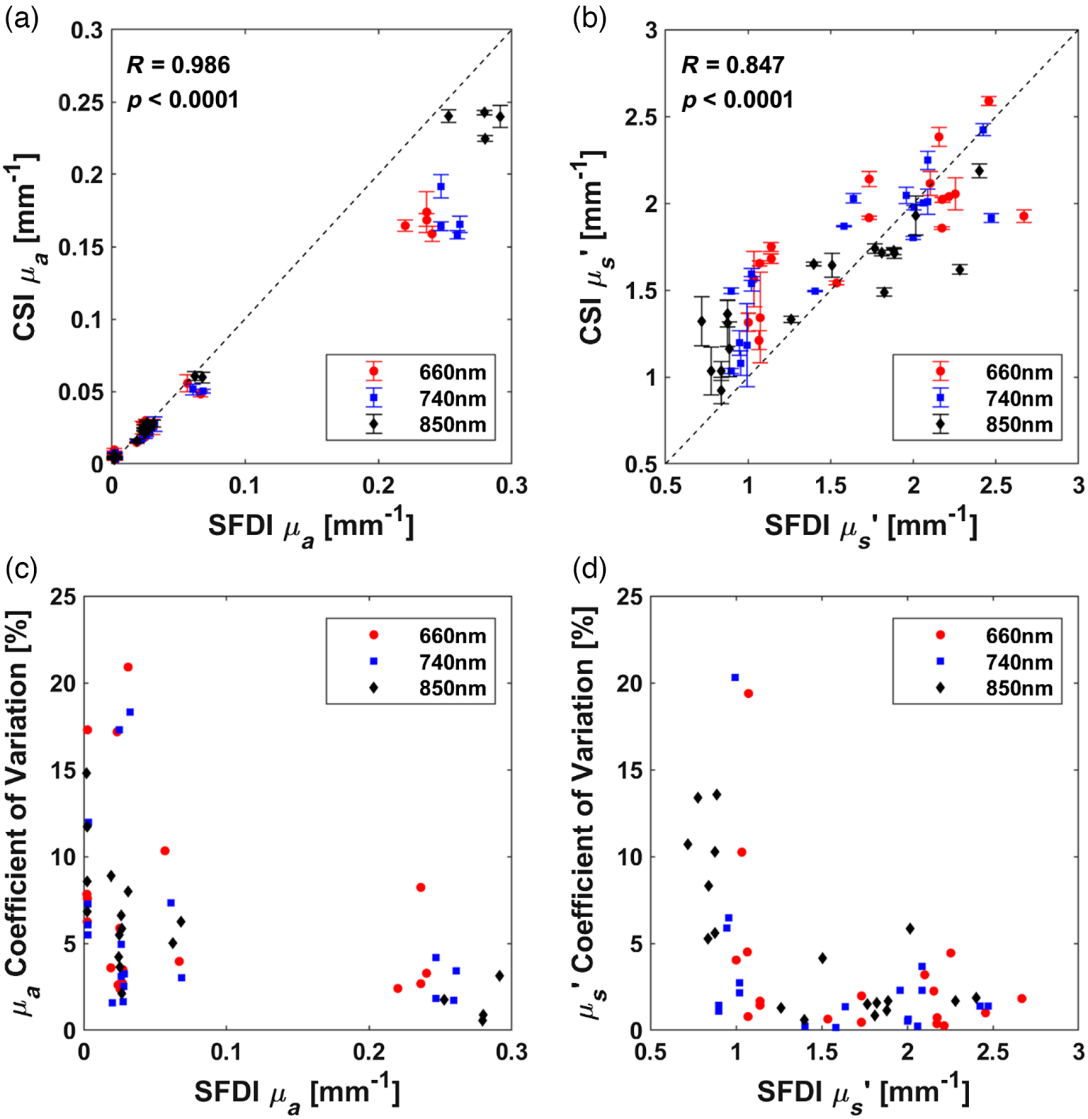
*In vitro* validation of CSI-measured optical properties compared to SFDI using 18 phantoms over 1 week. (a) Comparison between CSI- and SFDI-measured $\mu_{a}$ (R=0.986, p<0.0001). (b) Comparison between CSI- and SFDI-measured $\mu_{s}^{\prime }$ (R=0.847, p<0.0001). Standard deviation error bars are shown. (c) COV of $\mu_{a}$ over 1 week. (d) COV of $\mu_{s}^{\prime }$ over 1 week.

To test device repeatability over a wide range of optical properties, we imaged 18 silicone-based tissue phantoms with CSI three times over 1 week, comparing the optical properties measured by CSI to those measured by SFDI. The results showed good agreement for $\mu_{a}$ (R=0.986, p<0.0001) and $\mu_{s}^{\prime }$ (R=0.847, p<0.0001) using Pearson’s correlation coefficient [Figs. 2(a) and 2(b)]. Error bars are the standard deviation over the three measurements over 1 week. CSI underestimated higher $\mu_{a}$ (>0.2) and had high variability at lower $\mu_{s}^{\prime }$. The correlation coefficients presented here are slightly lower than previously presented by Petitdidier et al.^23^ This difference may be due to the simulated reflectance curves from the look-up table not incorporating the numerical aperture or the working distance between the sensor and the measured phantom. The average COV over 1 week for $\mu_{a}$ at 660, 740, and 850 nm were 8.4%, 9.9%, and 6.5%, respectively. The average COV for $\mu_{s}^{\prime }$ at 660, 740, and 850 nm were 4.0%, 4.6%, and 6.2%, respectively. The COVs demonstrate good repeatability, but low $\mu_{a}$ and $\mu_{s}^{\prime }$ had higher COVs [Figs. 2(c) and 2(d)], possibly due to a relatively long source–detector separation (3 to 10 mm) and low signal to noise at high $\mu_{a}$. Furthermore, the low $\mu_{a}$ phantoms ($\mu_{a}∼0.003\;{\mathrm{mm}}^{-1}$) had higher COVs, due to small differences in the recovered optical properties. Improved signal-to-noise and better repeatability may be possible using multiple exposure times for srDRS curves.^23^^,^^24^

To test long-term device repeatability, we imaged the same 18 silicone-based tissue phantoms nine times over 3 months. The average COVs for $\mu_{a}$ at 660, 740, and 850 nm were 19.3%, 20.7%, and 19.5%, respectively, whereas the average COVs for $\mu_{s}^{\prime }$ at the three wavelengths were 5.4%, 5.7%, and 6.1%, respectively. These findings indicate that over an extended period, the CSI device has twice the variability in $\mu_{a}$ compared with short-term results [Figs. 2(c) and 2(d)], whereas the variability in $\;\mu_{s}^{\prime }$ remained relatively constant.

**Fig. 3:**
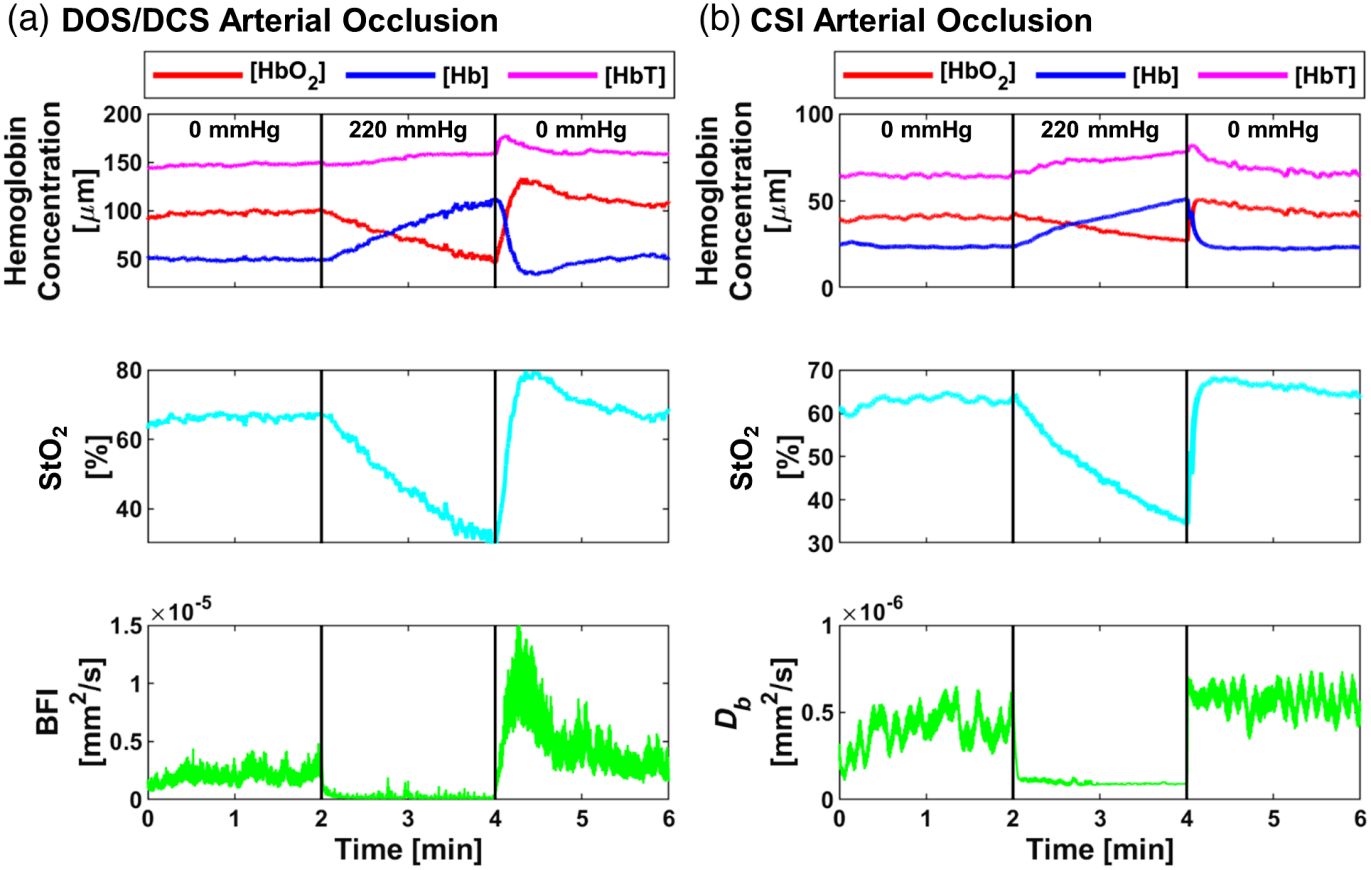
Representative *in vivo* validation from one subject of the CSI sensor compared to DOS/DCS during an arterial occlusion. (a) DOS/DCS arterial occlusion. (b) CSI arterial occlusion. An arterial occlusion protocol with a 2-min baseline period, followed by 2 min of full occlusion at 220 mmHg, and then 2 min of recovery. Abbreviations: oxy-hemoglobin concentration, $\left[{\mathrm{HbO}}_{2}\right]$; deoxy-hemoglobin concentration, [Hb]; total hemoglobin concentration, [HbT]; tissue oxygen saturation, ${\mathrm{StO}}_{2}$; blood flow index, BFI; and Brownian diffusion coefficient, $D_{b}$.

We compared CSI with DOS/DCS to evaluate their ability to assess *in vivo* hemodynamics. Both DOS/DCS [Fig. 3(a)] and CSI [Fig. 3(b)] showed similar trends in absolute hemodynamic measurements during an arterial occlusion. Blood flow rapidly decreased during the occlusion, leading to an increase in [Hb] and a decrease in $\left[{\mathrm{HbO}}_{2}\right]$ and ${\mathrm{StO}}_{2}$. However, after arterial occlusion release, DOS/DCS demonstrated a hyperemic response, whereas CSI returned to baseline levels. This difference could be attributed to the measurement location (finger versus thenar muscle) or the source–detector separation (3 to 10 mm versus 15 mm). We performed two additional arterial occlusion experiments to assess if the measurement location played a role in the difference between the DOS/DCS and CSI hyperemic responses. First, we used a finger-clip LSI-based device^7^^,^^8^ to measure blood flow from the end of the finger during an arterial occlusion. Second, we performed a large field-of-view (FOV) measurement of the hand with LSI and selected multiple ROIs, including the fingers and thenar muscle. The finger-clip measurement lacked a post-arterial occlusion hyperemic response (Fig. S1 in the Supplemental Material). The large FOV measurement showed that the tip of the finger had the smallest hyperemic response post-arterial occlusion (Fig. 2 in the Supplemental Material). These data suggest the lack of post-arterial hyperemic response is at least partially due to measurement location.

**Fig. 4:**
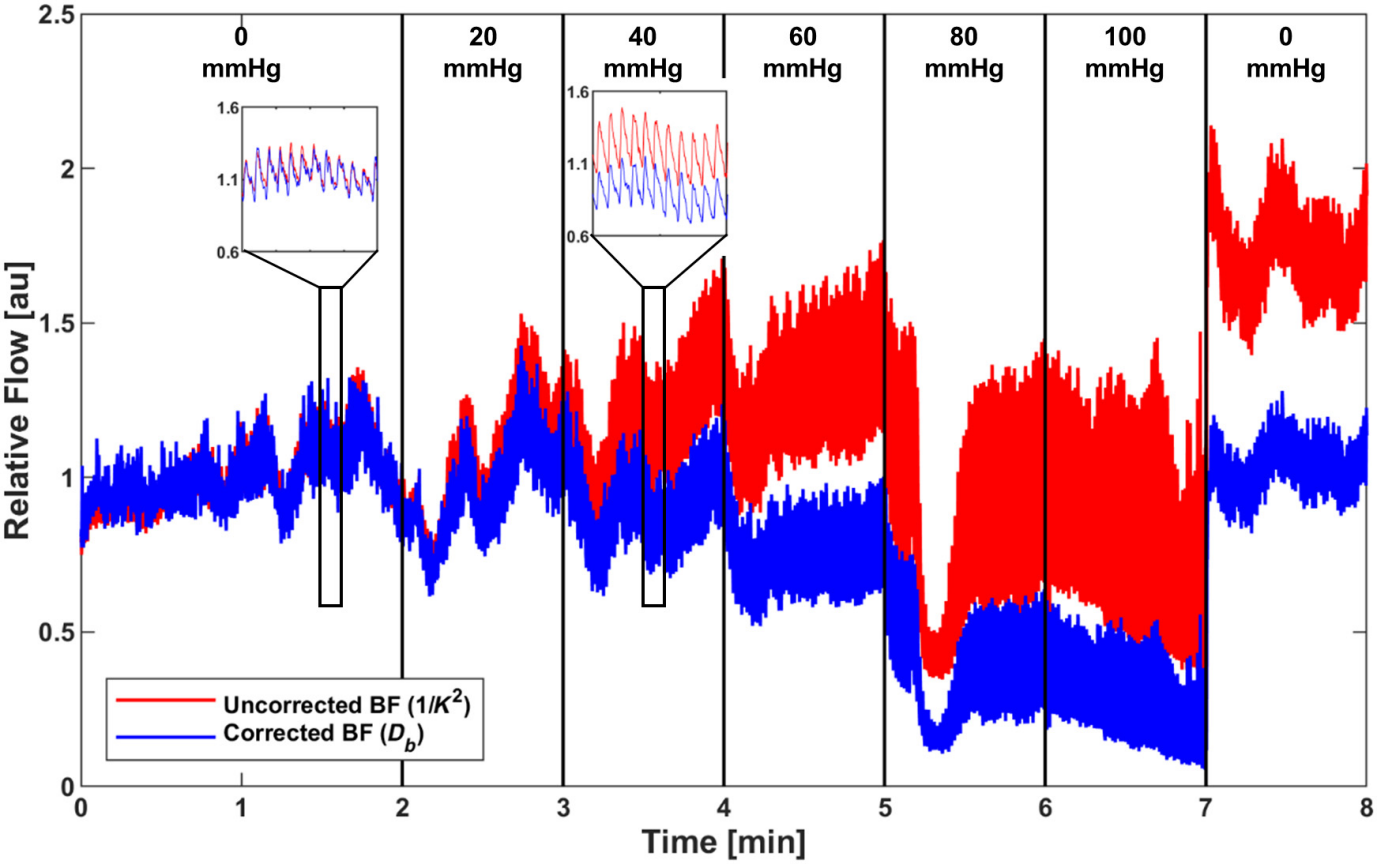
Representative comparison of uncorrected and optical property-corrected blood flow from one subject using the CSI sensor during venous occlusion. The venous occlusion protocol consisted of a 2 min baseline period, followed by a stepwise venous occlusion at 20, 40, 60, 80, and 100 mmHg, where each occlusion pressure lasted 1 min. After the 100 mmHg pressure, the pressure was released for a 1 min recovery period. Uncorrected blood flow is in red and corrected blood flow is in blue. Insets show pulsatile blood flow waveform at 0 and 40 mmHg.

We next compared uncorrected and optically corrected blood flow using venous occlusion data from the CSI device (Fig. 4). Both uncorrected and optically corrected blood flow showed a decreased trend as pressure increased, and the pulsatile blood flow signals (SPG) were clearly distinguishable and robust. However, a stark difference was observed during the venous occlusion: the optically corrected blood flow showed a ∼200% greater decrease compared with the uncorrected blood flow at 100 mmHg. These findings align with the previous studies that use improved mathematical models describing laser speckle fluctuations in dynamic light scattering, such as LSI.^29^ Specifically, LSI has been found to underestimate the decrease in cerebral blood flow by 300% in cases of ischemic stroke.^29^^,^^30^

**Fig. 5:**
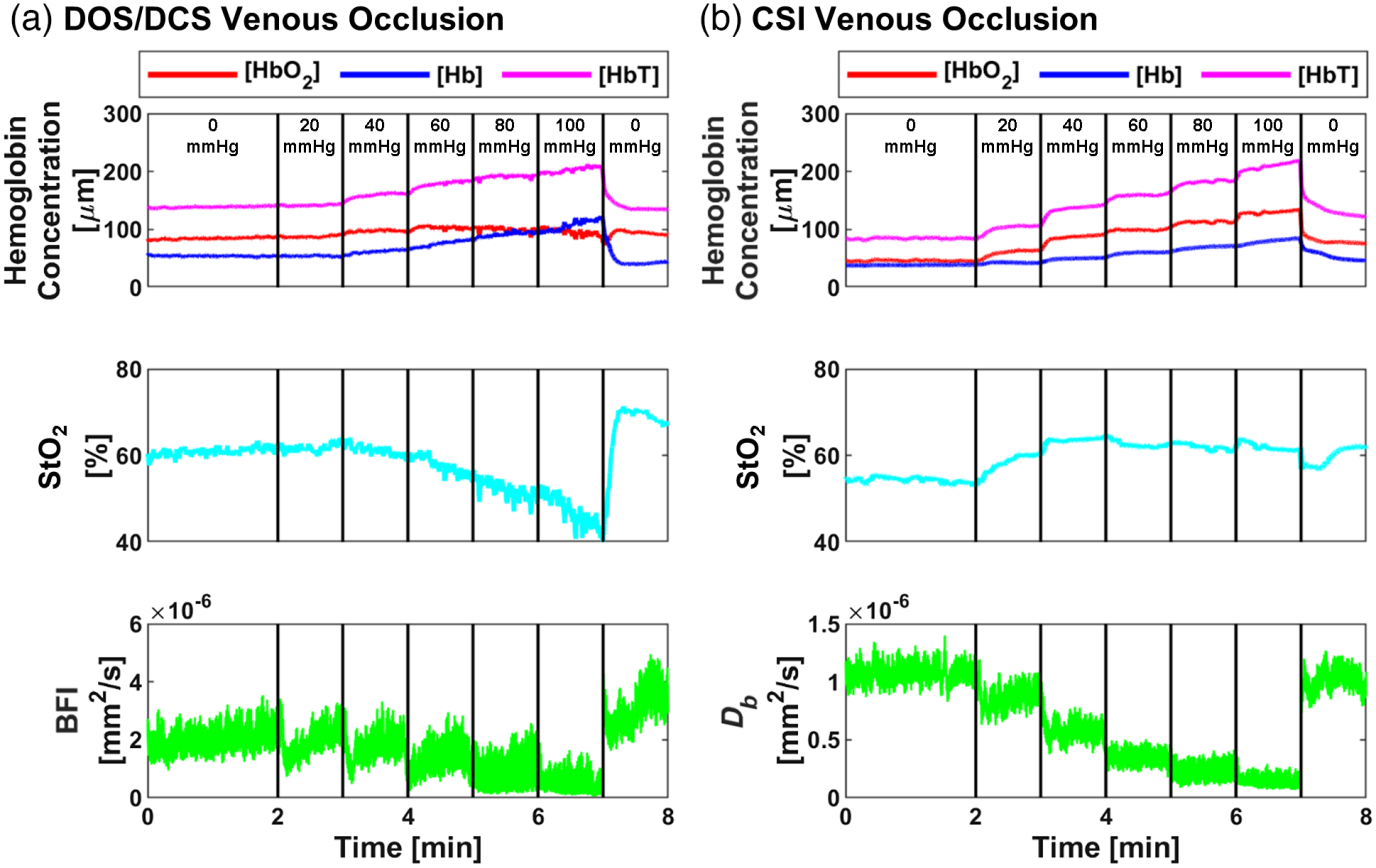
Representative *in vivo* validation from one subject of the CSI sensor compared with DOS/DCS during a venous occlusion. (a) DOS/DCS venous occlusion. (b) CSI venous occlusion. The venous occlusion protocol consisted of a 2 min baseline period, followed by a stepwise venous occlusion at 20, 40, 60, 80, and 100 mmHg, where each occlusion pressure lasted 1 min. After the 100 mmHg pressure, the pressure was released for a 1 min recovery period. Abbreviations: oxy-hemoglobin concentration, $\left[{\mathrm{HbO}}_{2}\right]$; deoxy-hemoglobin concentration, [Hb]; total hemoglobin concentration, [HbT]; tissue oxygen saturation, ${\mathrm{StO}}_{2}$; blood flow index, BFI; and Brownian diffusion coefficient, $D_{b}$.

To assess the ability of CSI to resolve *in vivo* hemodynamics, we compared CSI with DOS/DCS during a venous occlusion. We expected that as venous occlusion pressure increased, [HbT] and [Hb] would increase, $\left[{\mathrm{HbO}}_{2}\right]$ would remain constant, and ${\mathrm{StO}}_{2}$ and blood flow (BFI) would decrease. DOS/DCS data supported this trend, especially at higher occlusion pressures (>60  mmHg) [Fig. 5(a)]. However, CSI data exhibited a different behavior [Fig. 5(b)]. While [HbT] and [Hb] increased and blood flow ($D_{b}$) decreased, $\left[{\mathrm{HbO}}_{2}\right]$ increased, leading to relatively constant ${\mathrm{StO}}_{2}$ levels. Several factors might explain this discrepancy. First, the measurement location differed, with CSI measured from the finger and DOS/DCS from the thenar muscle on the palm. Second, the vascular compartment and sample volume differed, as DOS/DCS had a source–detector separation of 15 mm, whereas CSI used separations from 3 to 10 mm. Third, metabolic differences between the finger and the thenar muscle could have existed. The finger may consume less oxygen than the incoming blood flow, resulting in increased $\left[{\mathrm{HbO}}_{2}\right]$ in the CSI measurements. Despite the discrepancy between DOS/DCS and CSI, previous studies^31^^–^^33^ using optical methods have also reported an increased $\left[{\mathrm{HbO}}_{2}\right]$, supporting our findings.

## 4 Conclusions

In this paper, we aimed to measure $\mu_{a}$, $\mu_{s}^{\prime }$, $\left[{\mathrm{HbO}}_{2}\right]$, [Hb], ${\mathrm{StO}}_{2}$, and blood flow, into a compact device similar to current pulse oximeters. To achieve this aim, we developed CSI, which combines srDRS and LSI. We demonstrated the effectiveness of CSI in measuring optical properties using tissue-simulating phantoms. Moreover, we showed that CSI can track changes in blood flow during arterial and venous occlusions and accurately detect the SPG signal. The inclusion of additional information in a pulse oximetry format could potentially enhance the measurement of optically derived blood pressure,^34^ $S_{p}O_{2}$, and metabolism,^15^^,^^35^^,^^36^ enabling the assessment of hemodynamic dysfunction in diseased and injured individuals.

## Data Availability

Data will be made available to academic researchers upon reasonable request.
