# Supplementary material for: Quantifying tissue properties and absolute hemodynamics using coherent spatial imaging
Source: J Biomed Opt. 2023 Dec 19;28(12):127001. doi: 10.1117/1.JBO.28.12.127001 (PMC10730023; doi:10.1117/1.JBO.28.12.127001)
Supplement: Supplementary file 1 [file JBO_028_127001_SD001.pdf]

Supplemental Material

We performed two additional arterial occlusion experiments to assess if the measurement location played a role in the difference between the DOS/DCS and CSI hyperemic responses. First, we used a finger-clip LSI-based device[7,8] to measure blood flow from the end of the finger during an arterial occlusion. Second, we performed a large field-of-view measurement of the hand with LSI and selected multiple ROIs, including the fingers and thenar muscle.

The finger-clip measurement lacked a post-arterial occlusion hyperemic response (Supplemental Figure 1). The large FOV measurement showed that the tip of the finger had the smallest hyperemic response post-arterial occlusion (Supplemental Figure 2). These data suggest the lack of post-arterial hyperemic response is at least partially due to measurement location.

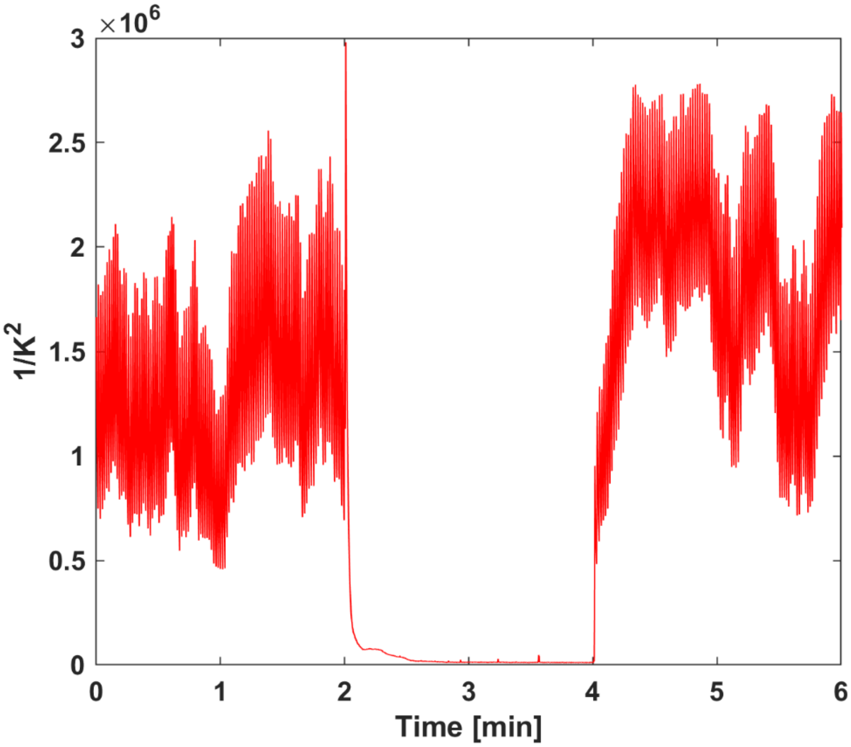

Supplemental Figure 1: Finger-clip transmittance LSI device does not show a hyperemic response post-arterial occlusion.

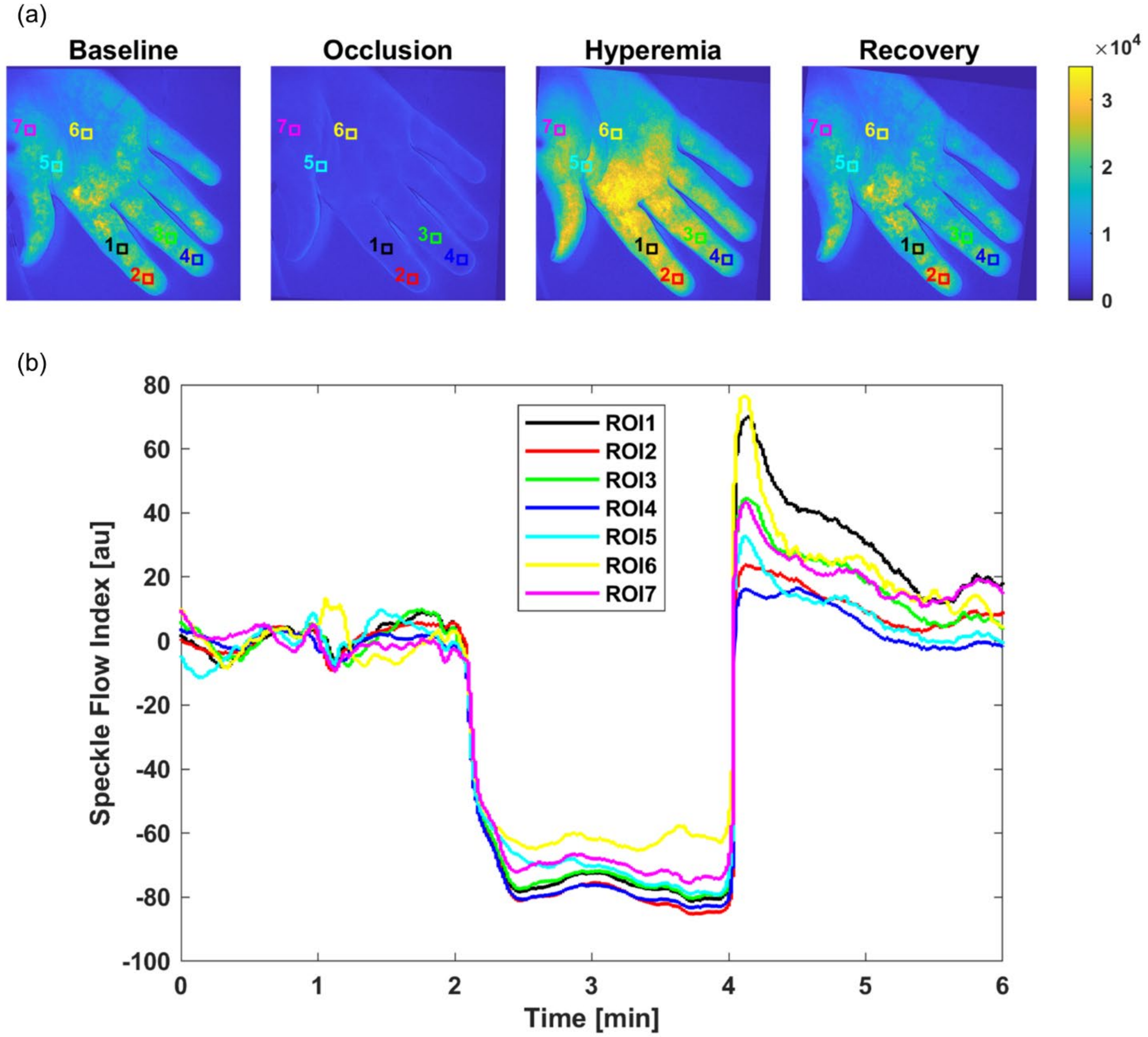

Supplemental Figure 2: Large field-of-view LSI setup shows that ROIs from the tip of the fingers (red and blue) have the smallest hyperemic response compared to other ROIs selected throughout the hand.
